# The Bacterial Response Regulator ArcA Uses a Diverse Binding Site Architecture to Regulate Carbon Oxidation Globally

**DOI:** 10.1371/journal.pgen.1003839

**Published:** 2013-10-17

**Authors:** Dan M. Park, Md. Sohail Akhtar, Aseem Z. Ansari, Robert Landick, Patricia J. Kiley

**Affiliations:** 1 Department of Biomolecular Chemistry, University of Wisconsin-Madison, Madison, Wisconsin, United States of America; 2 Department of Biochemistry, University of Wisconsin-Madison, Madison, Wisconsin, United States of America; 3 Department of Bacteriology; University of Wisconsin-Madison, Madison, Wisconsin, United States of America; 4 Great Lakes Bioenergy Research Center, University of Wisconsin-Madison, Madison, Wisconsin, United States of America; Institute of Molecular and Cell Biology (IMCB), A*STAR, Singapore

## Abstract

Despite the importance of maintaining redox homeostasis for cellular viability, how cells control redox balance globally is poorly understood. Here we provide new mechanistic insight into how the balance between reduced and oxidized electron carriers is regulated at the level of gene expression by mapping the regulon of the response regulator ArcA from *Escherichia coli*, which responds to the quinone/quinol redox couple via its membrane-bound sensor kinase, ArcB. Our genome-wide analysis reveals that ArcA reprograms metabolism under anaerobic conditions such that carbon oxidation pathways that recycle redox carriers via respiration are transcriptionally repressed by ArcA. We propose that this strategy favors use of catabolic pathways that recycle redox carriers via fermentation akin to lactate production in mammalian cells. Unexpectedly, bioinformatic analysis of the sequences bound by ArcA in ChIP-seq revealed that most ArcA binding sites contain additional direct repeat elements beyond the two required for binding an ArcA dimer. DNase I footprinting assays suggest that non-canonical arrangements of cis-regulatory modules dictate both the length and concentration-sensitive occupancy of DNA sites. We propose that this plasticity in ArcA binding site architecture provides both an efficient means of encoding binding sites for ArcA, σ^70^-RNAP and perhaps other transcription factors within the same narrow sequence space and an effective mechanism for global control of carbon metabolism to maintain redox homeostasis.

## Author Summary

The cofactor NAD^+^ plays a central role in energy conservation pathways, shuttling electrons from the oxidation of growth substrates to respiratory or fermentative pathways. To sustain catabolism and cellular ATP demand, an appropriate balance between the reduced and oxidized forms of NAD^+^ must be maintained. Our genome-scale analysis of the transcription factor ArcA provides insight into how this process is transcriptionally regulated in *E. coli* in the absence of O_2_. We found that ArcA mediates a previously unrealized comprehensive transcriptional repression of genes encoding proteins associated with oxidation of non-fermentable carbon sources. Through the repression of these pathways, oxidized NAD^+^ is effectively preserved for fermentation pathways, facilitating energy conservation and preserving a balance between the oxidized and reduced forms of NAD^+^ in the absence of aerobic respiration. In addition, we found that the majority of ArcA binding sites contain additional sequence elements beyond that required for binding of an ArcA dimer, providing novel insight into how ArcA and other members of the largest class of two component system-response regulators (OmpR/PhoB family) may achieve global regulation of gene expression.

## Introduction

Maintaining redox balance is a crucial function for cell survival. Alteration of the cellular redox environment has been shown to affect a broad range of biological processes including energy metabolism [1]–[3], protein folding [4], signaling and stress responses [5]–[9]. Despite this, we have only a superficial understanding of how cells control redox homeostasis at a global level. Since the cellular redox environment is a reflection of many different redox couples [10], some of which are linked together through enzymatic reactions, an improved understanding of this process requires knowledge of how the redox state of each couple is controlled. One such important redox couple is NADH/NAD^+^, which plays a central role in catabolic pathways, shuttling electrons between donor and acceptor molecules and allowing cells to convert energy from various reduced substrates into cellular ATP. To ensure that catabolism proceeds, a balance between the rates of oxidation and reduction of NAD^+^ must be maintained. Many diverse regulatory mechanisms have evolved amongst different organisms to control the redox state of the NADH/NAD^+^ couple [6], [11]–[14]. In this study we investigated transcriptional inputs into this process by mapping the regulon of the transcription factor ArcA in *Escherichia coli*.

The ArcAB two component system, comprised of the membrane bound sensor kinase, ArcB, and the response regulator, ArcA, coordinates changes in gene expression in response to changes in the respiratory and fermentative state of the cell [15], [16]. This system is maximally activated in *E. coli* under anaerobic fermentative conditions when NADH from central metabolism is recycled to NAD^+^ by formation of the end products succinate, ethanol and lactate. The DNA binding activity of ArcA is regulated through reversible phosphorylation by ArcB [17], whose kinase activity is governed by the redox states of the ubiquinone and menaquinone pools [18]–[20] that are linked to the NADH/NAD^+^ redox couple through respiration. In the absence of O_2_, decreased flux through the aerobic respiratory chain lowers the ratio of oxidized to reduced quinones, stimulating ArcB kinase activity and transphosphorylation of ArcA [19]. Additionally, fermentation products have been shown to enhance the rate of ArcB autophosphorylation [21] and there is a positive correlation between the rate of fermentation and the levels of phosphorylated ArcA (ArcA-P) [16]. Thus, enzymatic linkage of the NADH/NAD^+^ couple to the oxidation state of the quinone pool and the production of fermentation products provides a link between the redox state of the NADH/NAD^+^ couple and the activity of the ArcAB system. Indeed, artificial perturbation of the NADH/NAD^+^ ratio has been shown to alter ArcA activity [22].

Consistent with the role of the ArcAB system in redox regulation, the majority of known ArcA targets in *E. coli* are associated with aerobic respiratory metabolism. Under anaerobic conditions, ArcA-P directly represses the operons encoding enzymes of the TCA cycle (*gltA*, *icdA*, *sdhCDAB-sucABCD*, *mdh*, *lpdA*) [23]–[27], and for the β-oxidation of fatty acids (*fadH*, *fadBA*, *fadL*, *fadE*, *fadD*, *fadIJ*) [25], lactaldehyde (*aldA*)/lactate oxidation (*lldPRD*) [24], [28], and glycolate/glyoxylate oxidation (*glcC*, *glcDEFGBA*) [29]. In contrast, ArcA-P activates the expression of operons encoding three enzymes that are important for adapting to microaerobic or anaerobic environments [cytochrome bd oxidase (*cydAB*) [24], pyruvate formate lyase (*focA-pflB*) [30] and hydrogenase 1 (*hya*) [31]]. However, gene expression profiling analyses indicate that the ArcA regulon is more complex than originally expected, including genes encoding a wide variety of functions outside of redox metabolism [32], [33]. Salmon et al. [33] and Liu et al. [32] each identified >350 genes that were differentially expressed when *arcA* was deleted. However, there was only a minimal overlap between these datasets and it is unclear how many of these genes are direct *vs.* indirect targets of ArcA. Thus, although ArcA plays a prominent role in the anaerobic repression of genes that encode enzymes for aerobic respiratory metabolism, the full extent of the ArcA regulon remains unclear, preventing a comprehensive understanding of its physiological role.

Despite the identification of several ArcA binding regions by footprinting, the sequence determinants for ArcA DNA binding are also not well understood. This is in large part due to the unusually long length (30–60 plus bp) [23], [24], [26], [28]–[30] and degenerate nature of these sequences, which makes bioinformatic searches challenging. Nevertheless, a 15-bp site consisting of two tandem direct repeats has been proposed as the ArcA recognition site [34]. A similar motif has been derived for *Shewanella oneidensis* ArcA based on binding energy measurements for every possible permutation of a 15-bp site [35]. However, a 15-bp site is insufficient to explain the extended footprints, raising the question of whether additional sequence conservation beyond 15 bp is important for ArcA DNA binding and transcriptional regulation.

To determine the *in vivo* binding locations of ArcA in *E. coli* under anaerobic fermentative growth conditions, we utilized chromatin immunoprecipitation followed by sequencing (ChIP-seq) or hybridization to a microarray (ChIP-chip). Bioinformatic analyses of sequences corresponding to ArcA-enriched regions were used to predict individual ArcA binding sites and to search for a binding motif that could explain the large ArcA footprints. Novel ArcA binding site architectures were then validated by DNase I footprinting. Additionally, gene expression profiling was performed in *arcA^+^* and Δ*arcA* backgrounds to determine the effect of ArcA DNA binding on gene expression. This combination of genome-wide approaches provided insight into the mechanism of ArcA DNA binding and transcriptional regulation. These results also allowed us to identify additional operons under direct ArcA control, thereby providing a more complete understanding of the physiological role of ArcA in *E. coli*.

## Results

### Identification of the chromosomal binding locations of ArcA

**Figure 1:**
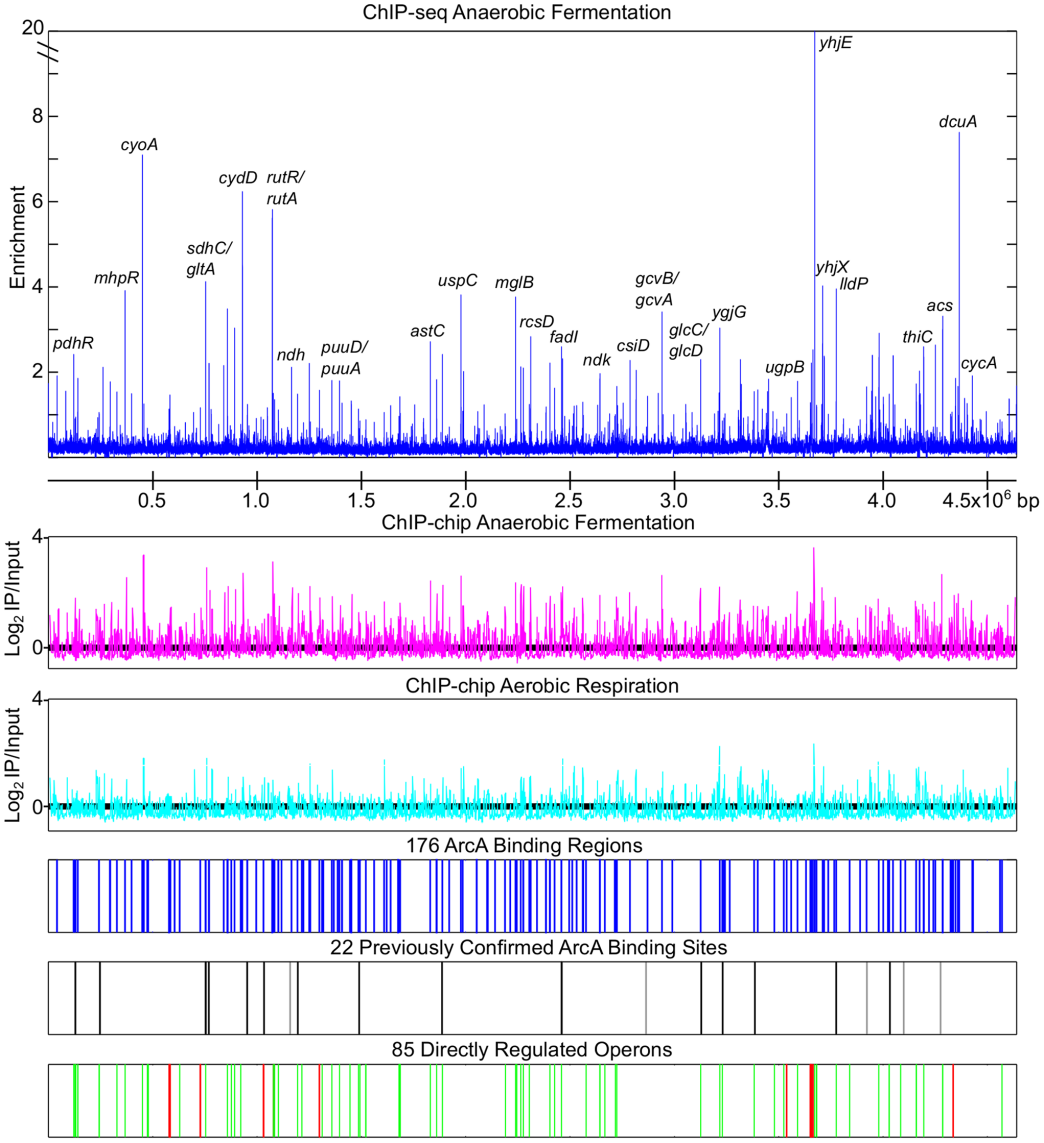
Genome wide overview of ArcA regulon analysis. The top panel depicts the anaerobic ArcA ChIP-seq data (blue) with a subset of peaks labeled. ArcA ChIP-chip data obtained from cells grown under anaerobic fermentation (magenta), or by aerobic respiration (cyan) are depicted using the same scale. Locations of the 176 ArcA binding sites identified by ChIP-chip and ChIP-seq are indicated by blue lines (Table S1). Locations of previously confirmed ArcA binding sites that were identified by our ChIP analysis are indicated with black lines while those not identified are indicated with grey lines. ArcA binding sites upstream of repressed operons (green lines) and activated operons (red lines) are depicted on the bottom tract (Table S6). Repression and activation were determined based on gene expression profiling in +*arcA* and Δ*arcA* strains (Table S5).

We mapped 176 chromosomal ArcA binding regions (Table S1) across the genome of *E. coli* K-12 MG1655 during anaerobic fermentation of glucose using ChIP-chip and ChIP-seq (Figure 1). These sites include all but five of the 22 previously identified ArcA binding regions (*uvrA/ssb* [36], *oriC* [37], *ptsG* [38], *rpoS* [39] and *sodA* [40]; Figure 1); the absence of a binding region upstream of *sodA* is likely the result of Fur outcompeting ArcA from binding [40]. ArcA binding was also examined during aerobic respiration using ChIP-chip and as expected, revealed a pronounced decrease in site occupancy (Figure 1) except for a handful of peaks (e.g., *ygjG* and *uxaB*), which were not investigated further. As ArcA protein levels remained relatively constant between aerobic and anaerobic conditions (data not shown and [16]), the decrease in occupancy under aerobic conditions can be explained by decreased ArcA-P levels, resulting from the increase in the ratio of oxidized to reduced quinones [20].

### ChIP-seq analysis provides improved resolution compared to ChIP-chip

**Figure 2:**
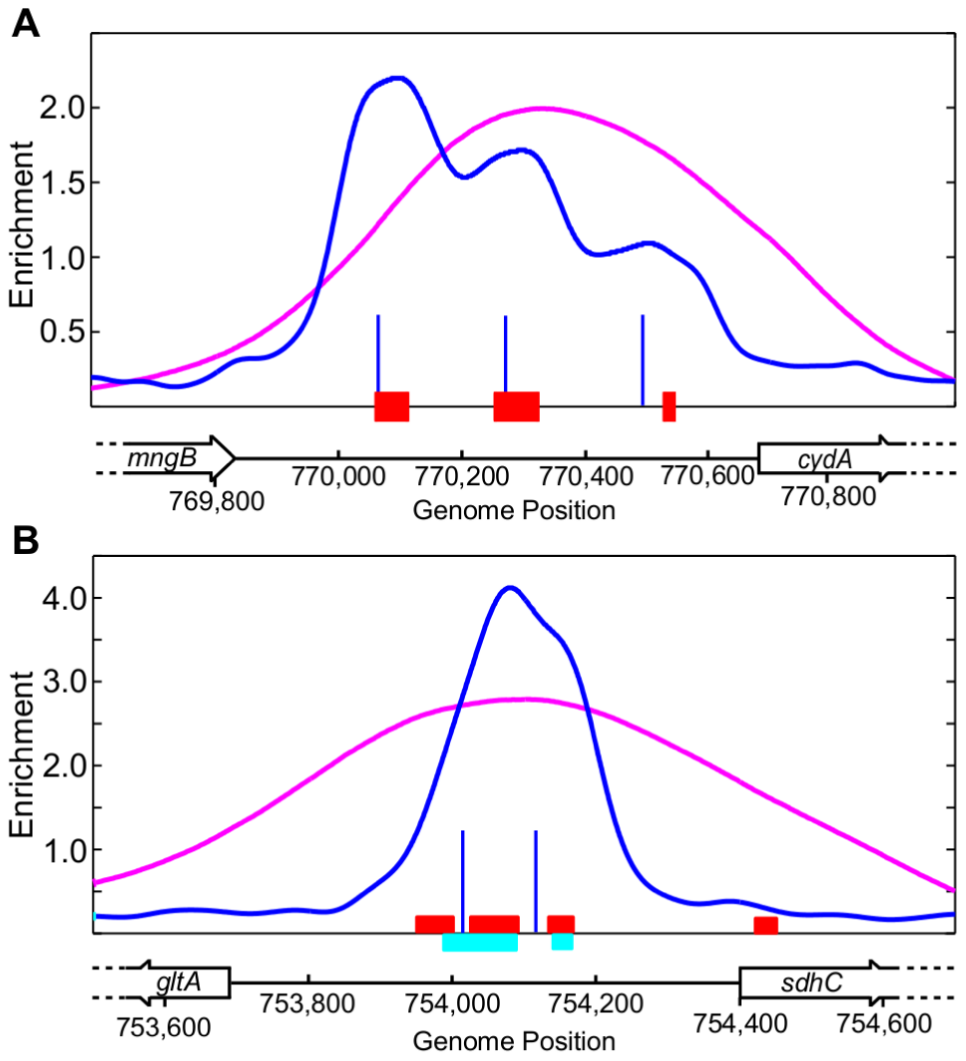
Examples of multiple ArcA binding regions identified within an intergenic region by ChIP-seq. (A) Three ArcA binding regions identified upstream of *cydA*. The ArcA ChIP-seq data and the CSDeconv-defined [41] binding locations are indicated by the blue trace and lines, respectively. The ChIP-chip trace is shown in magenta. Genes are represented by black boxes pointing in the direction of transcription. The previously determined ArcA footprint region is denoted by red boxes [24]. (B) Two ArcA binding regions identified within the *sdhC/gltA* divergent promoter region. The previously determined ArcA footprint regions are denoted by red [27] and cyan [24] boxes. See Table S2 for a list of all intergenic regions with multiple ArcA binding sites.

Overall, there was good agreement between the ChIP-chip and ChIP-seq datasets (109 peaks in common). However, 15 regions identified by ChIP-chip were resolved into 32 binding regions (Table S2) using ChIP-seq and the CSDeconv peak deconvolution algorithm [41]. For example, compared to only one binding region resolved with ChIP-chip, three binding regions were identified upstream of *cydA* (Figure 2A) and two were identified within the divergent *sdhC*/*gltA* (Figure 2B) promoter region using ChIP-seq. Furthermore, the position of the peak calls with CSDeconv is consistent with the position of known ArcA binding sites mapped by DNase I footprinting within these promoters [24], [27] and 29 of these 32 regions contain a predicted ArcA binding site (Table S2). The correlation of footprinted sites and predicted sites with CSDeconv peak calls allowed us to establish that binding sites separated by as little as 76 bp (based on the CSDeconv-defined coordinate for each binding region) could be resolved. From this analysis, several novel closely spaced ArcA binding sites, e.g. three binding regions upstream of *cyo* and two binding regions upstream of *nuo* and *pdhR-aceEF-lpdA*, were identified. Thus, since ChIP-seq provided higher resolution identification of ArcA binding sites, this dataset was used for all other analyses.

### More than 50% of ArcA binding sites have additional DR elements beyond the ArcA box

**Figure 3:**
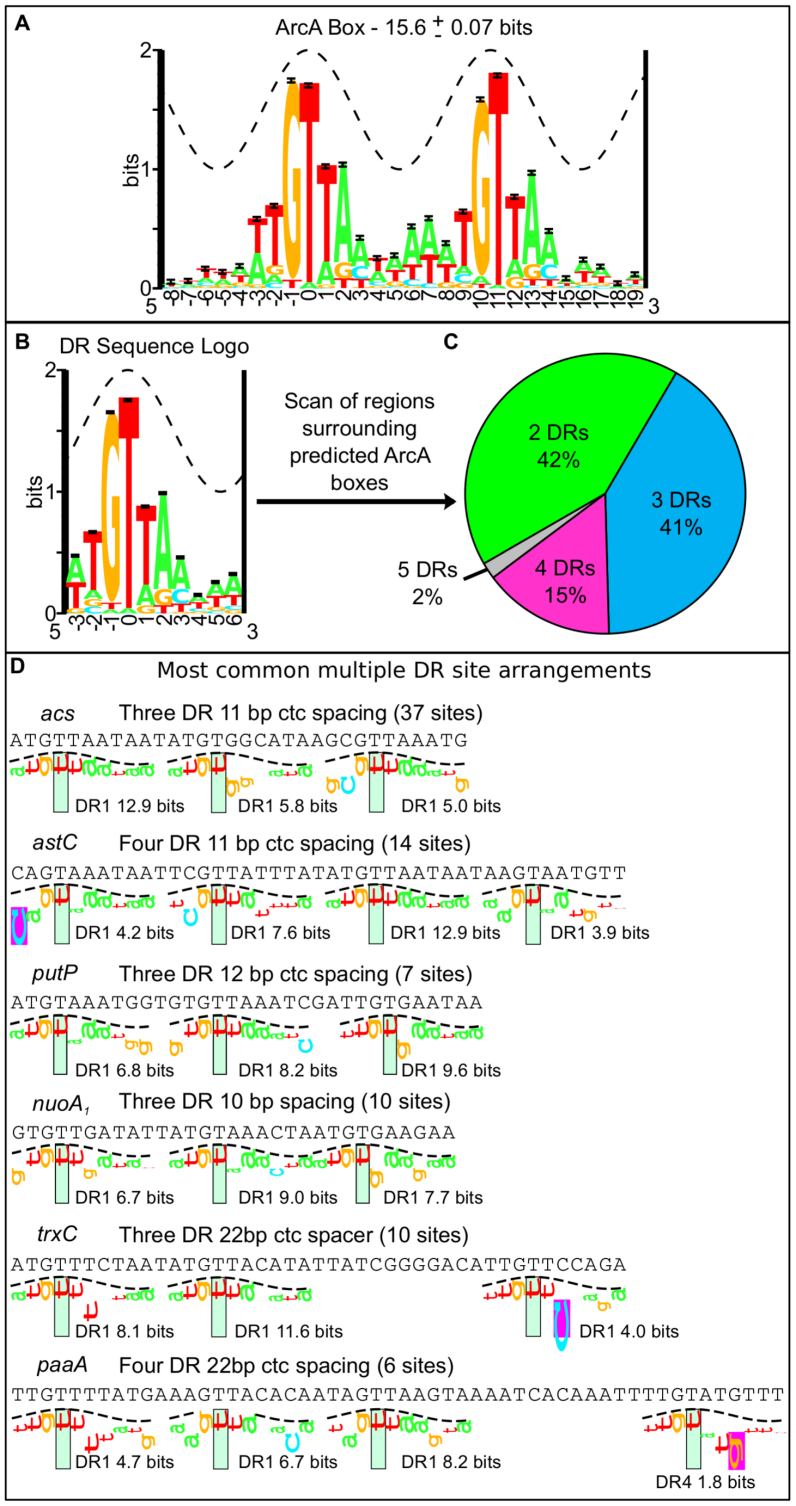
Bioinformatic analysis of the sequence regions bound by ArcA *in vivo*. (A) The 18-bp ArcA box sequence logo was constructed from the alignment of 128 ArcA boxes identified with a motif search of the 146 regions bound by ArcA in both ChIP-seq replicates (Table S3). The sequence conservation (bits) is depicted by the height of the letters with the relative frequencies of each base depicted by its relative heights [113]. The total sequence conservation is 15.6±0.07 bits in the range from positions −3 to +14. The crest of the sine wave represents the major groove of B-form DNA. (B) Sequence logo for a single direct repeat element. The total sequence conservation is 7.6±0.03 bits in the range from −3 to +6. The sequence regions surrounding each ArcA box (158) were scanned with this 10 bp PWM. (C) The distribution of two, three, four and five direct repeat binding sites in the regions bound by ArcA *in vivo* (See also Table S4). (D) Examples of some common multiple DR sites displayed using sequence walkers [100]. The number of sites with this same binding site architecture is listed in parentheses and the Ri (bits) for each DR element is indicated under the sequence walker.

DNase I footprinting experiments indicate that ArcA-P typically binds to long stretches of DNA (30–60+ bp) [23], [24], [26], [28]–[30]. However, the sequence determinants beyond a 15 bp direct repeat within these long stretches are not well understood. Using our high resolution binding regions, we searched for a common sequence recognition element [42], which identified a 18-bp sequence motif consisting of two direct repeat (DR) elements with a center to center (ctc) distance of 11 bp, close to the 10.5 bp per helical turn of B-form DNA, in nearly every (158 of 176) ArcA binding region (Figure 3A; Table S3). While this result extended the previously described ArcA box from 15 to 18 bp [34], we also found that many sites contained additional DR elements beyond the two DRs of the ArcA box. We then systematically searched the sequences surrounding each ArcA box with a 10-bp pair weight matrix (PWM), corresponding to a single DR element (Figure 3B), which revealed a diversity in the number and spacing of DR elements within ArcA binding sites. Although the largest class of binding sites contained just two DR elements at a ctc spacing of 11 bp (66), the majority of ArcA-binding sites (92) contain three to five DR elements predominantly at a ctc spacing of 11 bp (Figure 3C–D, Table S4).

**Figure 4:**
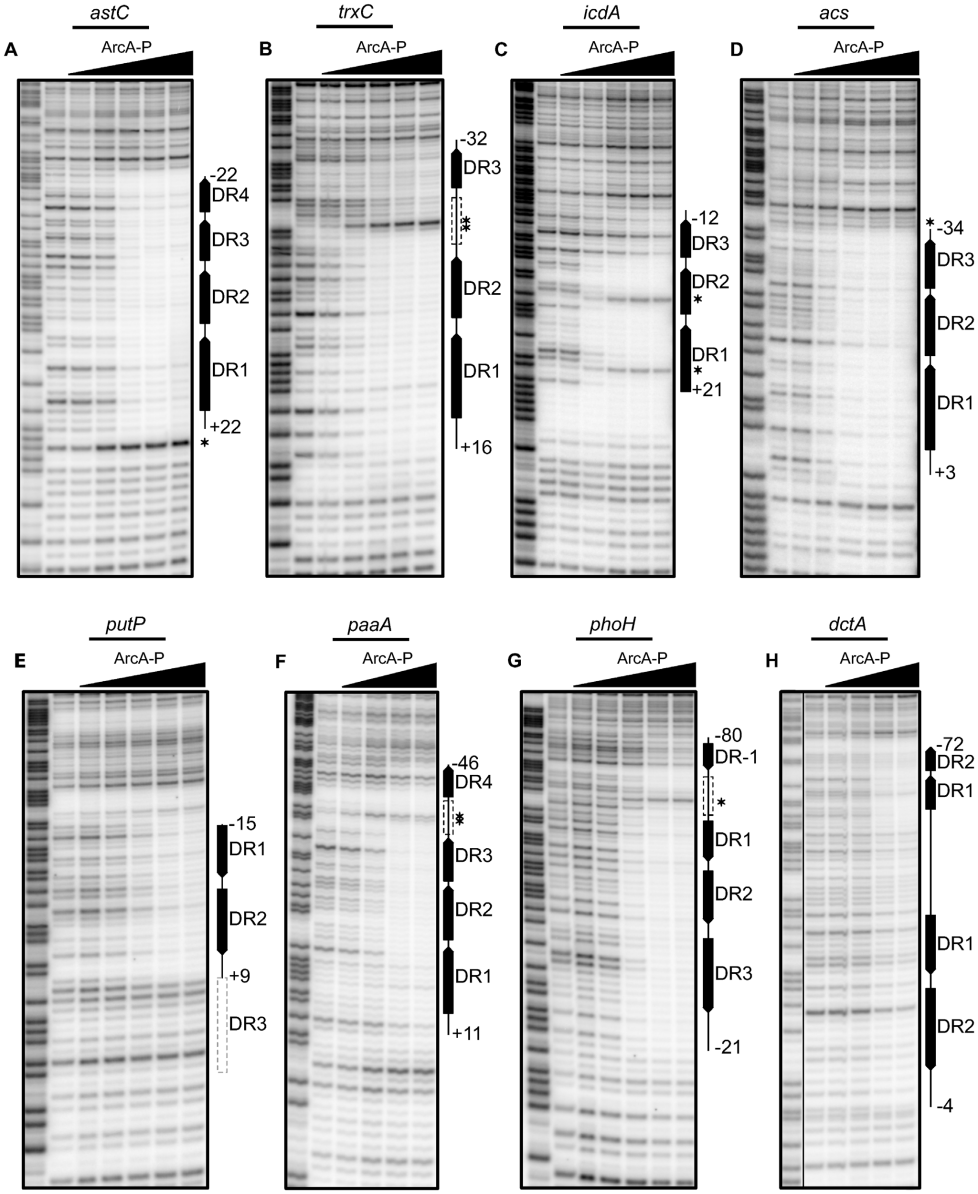
Analysis of predicted multiple DR elements by DNase I footprinting. DNase I footprinting data for a subset of ArcA regulated promoters. The regions protected by ArcA-P are indicated with vertical lines with predicted DR elements indicated by filled boxes with arrows indicating the directional orientation of DR elements. The numbers indicate the position relative to the previously determined transcription start site. Predicted DR elements not protected by ArcA-P are indicated by dashed grey boxes while dashed black boxes represent protected regions where no DR element greater than 0 bits was predicted. Samples were electrophoresed with Maxam–Gilbert ladders (A+G) made using the same DNA (lane 1). ArcA-P protein concentrations are given from left to right in terms of nM total protein. (A) Coding strand of the *astC* promoter, (D) *acs* promoter, (E) *putP* promoter and (G) *phoH* promoter. ArcA-P: 0, 50, 100, 200, 400, 600 nM. (B) Coding strand of the *trxC* promoter and (H) *dctA* promoter. ArcA-P: 0, 100, 200, 400, 600, 1000 nM. (C) Coding strand of the *icdA* promoter. ArcA-P: 0, 50, 150, 300, 600, 1000 nM. (F) Coding strand of the *paaA* promoter. ArcA-P: 0, 100, 200, 400, 600 nM.

To validate the bioinformatic predictions, DNase I footprinting was performed for a representative set of promoters. Since the OmpR/PhoB family of response regulators is expected to dimerize upon phosphorylation [43], we hypothesized that ArcA would bind as two adjacent dimers to sites with three consecutive DR elements (e.g., *icdA* and *acs*), three DR elements at which the distal DR is separated from DR2 by approximately two helical turns (22 bp; e.g., *trxC*), or four consecutive DR elements (e.g., *astC*) and in each case, protect a region the size of four DRs (∼44 bp). As anticipated, ArcA-P protected a 44 bp region at the *astC* promoter (Figure 4A) and a 48 bp region at the *trxC* promoter (Figure 4B). In contrast, ArcA-P only protected 33 bp and 37 bp regions, respectively, at the *icdA* and *acs* promoters, which encompassed the three consecutive DR elements (Figure 4C–D). The result for *icdA* is in agreement with previous footprinting data [23].

Our footprinting data also suggested that the spacing between DR2 and DR3 is likely important for ArcA-P binding, because ArcA-P did not protect a predicted DR3 element in which the ctc distance between DR2 and DR3 contained an extra bp (12-bp spacing; *putP*); protection corresponded to only DR1 and DR2 (Figure 4E). A potential explanation of this result is that the increased spacer distance disrupted potential protein-protein contacts between ArcA dimers. Additionally, our footprinting data identified 57 bp and 60 bp ArcA-P-binding regions, respectively, at the *paaA* and *phoH* promoters, which spanned from three consecutive predicted DRs to a distal DR element spaced nearly two full helical turns away (22 bp) (Figure 4F–G). As expected, no footprints were detected with unphosphorylated ArcA (data not shown).

Unexpectedly, the ArcA-P footprint at the *dctA* promoter extended 50 bp downstream of the predicted two DR site (Figure 4H), although this extended region was less well protected. A bioinformatic search revealed a second, but weaker two DR site at the downstream end of this protected region on the opposite DNA strand but no DR elements in the intervening 24 bp region, suggesting that protein-protein contacts may compensate for the absence of identifiable sequence elements at this site. Altogether, these results suggest that the length of the ArcA-P footprint reflects the location of the outermost DR elements within the binding site. In addition, these data reveal plasticity in the architecture among ArcA binding sites with anywhere from two to five DR elements of differing predicted strength present at any given site.

The footprinting results also revealed interesting features about ArcA-P DNA binding. At *acs* and *astC*, all DR elements were occupied at the same ArcA-P concentration, whereas at *icdA*, *paaA*, *phoH*, and *trxC*, occupation of DR3 or DR4 required a higher concentration of ArcA-P. The difference in concentration dependent occupancy of the DR elements at the *icdA* and *acs* promoters likely reflects the fact that DR3 of *acs* is a better match to the ArcA DR element PWM than DR3 of *icdA* (5 bits versus 3 bits). Furthermore, the transition from an unbound to bound state occurred over a narrow range in ArcA-P concentration, suggesting that ArcA-P binding to DR sites is cooperative, although the apparent degree of cooperativity also varied from site to site. Cooperative binding was particularly striking at the *acs* and *astC* promoters and for the three DR region at the *phoH* promoter, for which saturation occurred with less than a four-fold increase in ArcA-P levels. Finally, we also found that the average sequence conservation of DR elements in predicted binding sites with two, three and four equally spaced DR elements decreases with an increasing number of repeats (Figure S1).

DNase I hypersensitive sites were observed at six of the tested promoters, suggesting that ArcA-P binding to multiple DR sites also results in a bend or kink in the DNA. However, the locations of these hypersensitive sites differed from site to site. For example, a hypersensitive site was observed within the spacer region between the 22-bp spaced DR element and the other DR elements at the *trxC*, *paaA* and *phoH* promoters, whereas hypersensitive sites were observed within DR1 and DR2 at the *icdA* promoter (+8 and +19). In contrast, hypersensitive sites were located upstream and downstream of the footprinted regions at the *acs* and *astC* promoters, respectively. Thus, the binding site architecture appears not only to dictate the length of ArcA-P binding sites, but also to affect the concentration dependence of site occupancy and the DNA structure at target operons. These variations in ArcA-P binding likely have important implications for global transcriptional regulation.

### ArcA-P directly regulates the expression of 85 operons under anaerobic fermentative growth conditions

**Figure 5:**
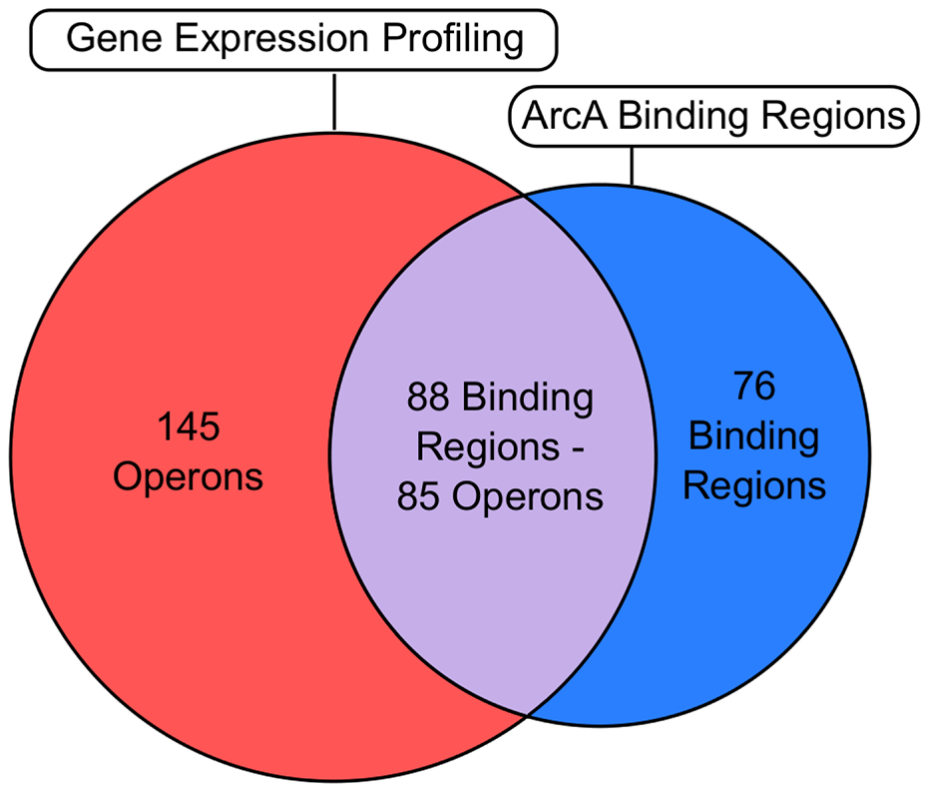
Correlation of the global binding site data with transcriptomic data. Venn diagram comparing ChIP-seq/ChIP-chip data (Table S1) with gene expression profiling data (Table S5). The overlap (grey) denotes operons that are directly regulated by ArcA (Table S6). The number of operons and binding regions in the direct regulon category are not equal because some directly repressed operons have multiple upstream ArcA binding regions (e.g., *cyo*), while in other cases, a single ArcA binding region is upstream of differentially expressed divergent operons (e.g., *glcC/glcD*). The magenta section represents the indirect regulon while the cyan section represents intergenic binding regions upstream of operons which did not exhibit differential expression under our growth conditions. Intragenic binding regions that were not upstream of differentially expressed operons were not included in this comparison.

To determine which ArcA binding regions exert an effect on transcription, genome-wide mRNA expression profiles for wild type (WT) and Δ*arcA* strains were examined. In total, 229 differentially expressed operons (Table S5) were identified, 85 of which were associated with one or more of 88 ArcA binding regions (Text S1) and, thus, are directly regulated by ArcA (Figure 5, Table S6). More than half of the operons that we found to be regulated directly by ArcA have not been previously reported (Table S6) but consistent with previous studies, ArcA acted predominantly as a transcriptional repressor (Figure 1).

#### ArcA directly represses 74 operons

**Figure 6:**
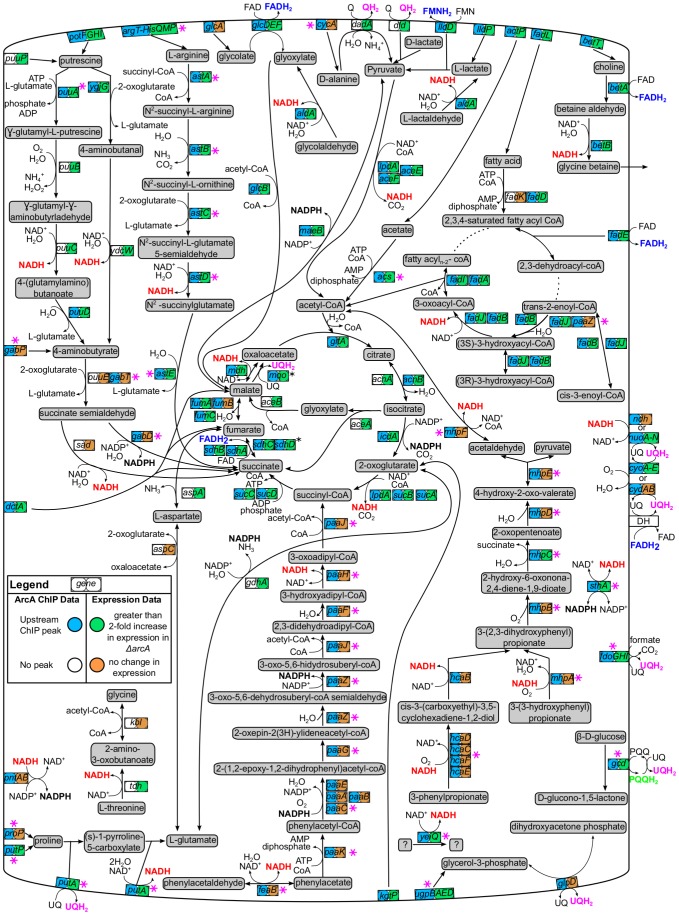
Carbon oxidation pathways regulated by ArcA. Pathway diagram depicting ArcA-repressed carbon oxidation pathways that would function during aerobic respiration. The genes encoding each pathway enzyme or transporter are represented by a box with two circles. ChIP-seq/ChIP-chip (first circle) and gene expression profiling data (second circle) were mapped using ProMeTra [114] with the colors described in the legend. Pink asterisks indicate novel directly regulated operons while reducing equivalents produced by ArcA repressed pathways are colored red (NADH), blue (FADH_2_), pink (QH_2_), black (NADPH) or green (PQQH). Not all pathway connections were included in order to improve clarity. Some operons encoding membrane-bound enzymes are depicted in the cytoplasm for clarity (black asterisks; e.g., *sdh*). Pathway information was obtained from EcoCyc [47].

ArcA functions predominantly as a global repressor of pathways associated with the oxidation of non-glycolytic carbon sources. This includes all previously identified ArcA targets associated with central metabolism (e.g., the genes encoding pyruvate dehydrogenase, cytochrome o ubiquinol oxidase, NADH-quinone oxidoreductase I, and the enzymes of the TCA cycle) (Figure 6). In addition, ArcA repressed the genes encoding enzymes, transcriptional regulators, or transporters associated with short chain acid/aldehyde oxidation (*aldA, lldPRD,* ***acs-yjcH-actP*** *, glcC, glcDEFGBA and* ***fdoGHI***; bolded operons have not been previously reported), amino acid and polyamine oxidation (*puuA, puuDR,* ***ygjG*** *, potFGHI,* ***astABCDE*** *,* ***argT-hisQMP*** *,* ***putA*** *,* ***putP***), β-oxidation of fatty acids (*fadH, fadBA, fadL, fadE, fadD, fadIJ,* ***tesB***), aromatic compound oxidation (*hcaR,* ***mhpR*** *,* ***feaR***), other carbon oxidation pathways (*betIBA, betT,* ***ugpBAED*** *,* ***gcd*** *,* ***maeB***) and peptide utilization (***cstA***). Other ArcA repressed targets include methionine sulfoxide reductase (***msrB***), thioredoxin 2 (***trxC***) [44], [45], a soluble pyridine nucleotide transhydrogenase (***sthA***) that reduces NAD^+^ with NADPH, and an ADP-sugar pyrophosphorylase (***nudE***) that could play a role in maintaining an optimal NADH/NAD^+^ ratio based on its ability to use NADH as a substrate [46]. Finally, an ArcA-regulated ribonucleoside transporter (***nepI***) and a nucleoside diphosphate kinase (*ndk*) could also function in NAD^+^ homeostasis via their functions in nucleotide metabolism [47].

A few repressed operons (9) encode proteins with functions not known to be associated with redox metabolism. This includes ***bssR*** and *csgD*, which encode transcription factors involved in biofilm formation and curli biosynthesis, respectively, and ***rsd***, encoding a stationary phase induced anti-σ factor. Additionally, ArcA repressed outer membrane proteins (***cirA***, ***ompW***), *a* potassium efflux system (*kefGB-yheV)*, the ATPase component of the ClpAP protease (***clpA***), an ATP binding protein (***phoH***) and a methyl-galactoside ABC transporter (***mgl***). Although a rationalization for ArcA repression of each of these operons is not yet known, the control of *mgl* may be related to the report that *E. coli* K-12 is unable to grow fermentatively on galactose [48]. Finally, 13 repressed operons have only predicted or unknown function [47]; four are predicted membrane proteins, two are predicted transcriptional regulators (***ydcI*** **,** ***yjiR***), and two others are predicted to encode a dehydrogenase (***yeiQ***) and a fimbrial-like adhesin protein (***yehD***), respectively.

**Figure 7:**
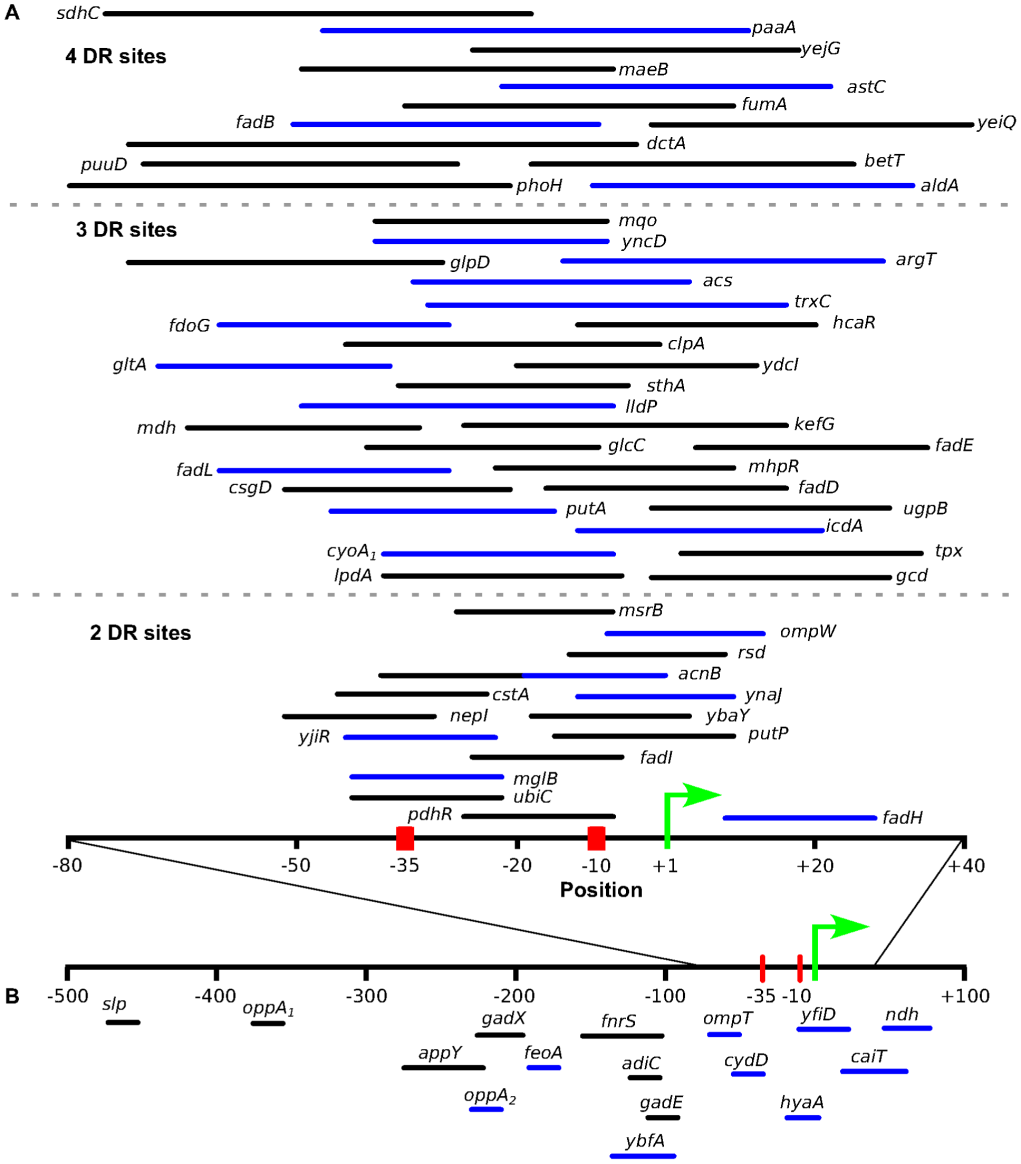
The location of predicted ArcA binding sites with respect to the known transcription start site. Location of predicted ArcA binding sites with respect to the experimentally determined TSS ([47], [50]) for directly repressed (A) and directly activated (B) operons. The positions used in this plot are listed in Table S6. Binding sites upstream of operons exhibiting differential expression in previous studies were also included (Table S7). For the repressed operons, ArcA binding sites are grouped based on the number of DR elements (separated by broken grey lines) and only sites which overlap the σ^70^-RNAP binding site were included. The length of the line is representative of the length of the binding site with the line color denoting a directional orientation on the coding strand (black) or noncoding strand (blue). The ArcA binding sites for *cydA* and *focA* were not included because they contain multiple TSS.

To gain insight into the mechanism of ArcA repression, we examined σ^70^ ChIP-seq data collected from growth conditions identical to those used with ArcA [49]. The vast majority (56/65) of ArcA-repressed promoters, exhibited a statistically significant reduction in σ^70^ peak height under anaerobic conditions compared to aerobic conditions, consistent with ArcA preventing RNA polymerase binding (Table S6). In agreement with this observation, correlation of the position of predicted ArcA binding sites with known σ^70^-dependent transcription start site (TSSs from EcoCyc [47] or [50]) indicated that the majority of repressed targets (52/66) with a confirmed TSS have an ArcA binding site that overlaps the region bound by σ^70^-RNAP (the TSS, the −35 element or the −10 elements; Figure 7A, Table S6). Eight promoter regions for ArcA-repressed operons did not exhibit a decrease in σ^70^ occupancy. Because these sites are located within divergently transcribed regions where the other operon is not affected by ArcA, σ^70^ occupancy may reflect only the adjacent non-ArcA-regulated promoter. In summary, the positioning of ArcA binding sites is consistent with the O_2_-dependent decrease in σ^70^ occupancy that is observed at nearly all ArcA-repressed operons, suggesting that ArcA likely represses transcription through promoter occlusion.

#### ArcA directly activates 11 operons

Analysis of the function of directly activated genes indicated a diversity of functions. This includes hydrogenase 1 (*hyaABCDEF*) [51], the ferrous iron transporter (***feoABC***), an oligopeptide ABC transporter (*oppA*), and the acid phosphatase transcriptional regulator (*appY*) that is involved in anaerobic gene regulation [52]. Our data also suggest a role for ArcA in the acid resistance response by activating operons encoding regulators of the glutamate dependent acid resistance system (*gadE-mdtEF* and *gadXW*) [53], [54], the arginine dependent acid resistance (***adiC***) system [55] and the resistance to organic acid stress (***slp-dctR***) [56]. The remaining ArcA-activated targets encode genes of unknown function (***ybcW*** and ***ybfA***) and a small regulatory RNA, *fnrS* [57], [58]. Although *fnrS* was not present on our microarrays, a previous study showed that ArcA is a coactivator of this sRNA [57].

Examination of the σ^70^ occupancy data indicated that there is a statistically significant change in σ^70^-RNAP occupancy under anaerobic conditions for nine of the 10 directly activated operons, consistent with ArcA functioning in activation of these operons (Table S6). However, both the position and orientation of the predicted ArcA binding site relative to the TSS for each operon is variable among activated targets (Figure 7B). Some binding sites are located downstream of the nearest mapped TSS, whereas others overlap the promoter elements or are located as far as 200–400 bp upstream of the TSS. Given this variable positioning and orientation of ArcA binding sites, it remains unclear whether ArcA can activate transcription by directly contacting σ^70^-RNAP as found with some OmpR/PhoB family members [59]–[61].

### The direct regulon of ArcA extends beyond the 85 operons identified under our growth conditions

Many intergenic ArcA binding regions (76) were associated with operons that did not show an ArcA dependent change in gene expression in our studies. However, previous studies indicated that 13 operons are regulated by ArcA but under different growth conditions (Table S7). For example, *cydAB* expression is activated by ArcA under microaerobic growth conditions, when FNR repression is relieved [62]. Furthermore, many binding regions (31) are associated with operons that are poorly expressed under our growth conditions in both the *arcA^+^* and *ΔarcA* strains (e.g., *paa* operon; Table S8). Since ArcA is predominantly a repressor of transcription, we hypothesized that these promoters were repressed by a second transcription factor or require a transcriptional activator and, therefore, growth under inducing conditions would be required to see an effect of ArcA binding on the transcription of these operons.

To test this idea, we constructed a *paaA* promoter-*lacZ* fusion and measured β-galactosidase activity in WT and *ΔarcA* strains supplemented with phenylacetate (PA) because the *paaABCDEFGHIJK* operon is known to be repressed by PaaX in the absence of PA [63]. In the presence of PA, ArcA strongly repressed *paaA-lacZ* expression under anaerobic conditions (23 Miller units for WT), whereas repression was relieved in a strain lacking ArcA (404 Miller units) or under aerobic conditions (294 and 372 Miller units for WT and *ΔarcA*, respectively), indicating that ArcA prevents induction of the *paa* operon under anaerobic conditions even when PA is present. Examination of regulatory data in EcoCyc [47] indicated that 11 other poorly expressed operons also are associated with other annotated activators or repressors (Table S8) that may contribute to synergistic regulation with ArcA. Furthermore, ChIP-chip experiments for other transcriptional repressors indicated that under our growth conditions, 15 targets are also bound by Fur, H-NS, or both [Beauchene and Kiley, personal communication; [49]] (Table S8). Thus, repression by Fur and H-NS may mask effects of ArcA. Altogether, these results indicate that ArcA repression likely serves as a secondary layer of control at many of these operons, ensuring that induction does not occur under anaerobic conditions even when the specific inducer is encountered. Thus, the 85 operons that show a change in expression under fermentative growth with glucose represent just a subset of the complete ArcA direct regulon.

### The indirect regulon of ArcA may reflect a hierarchical mode of transcriptional regulation

**Figure 8:**
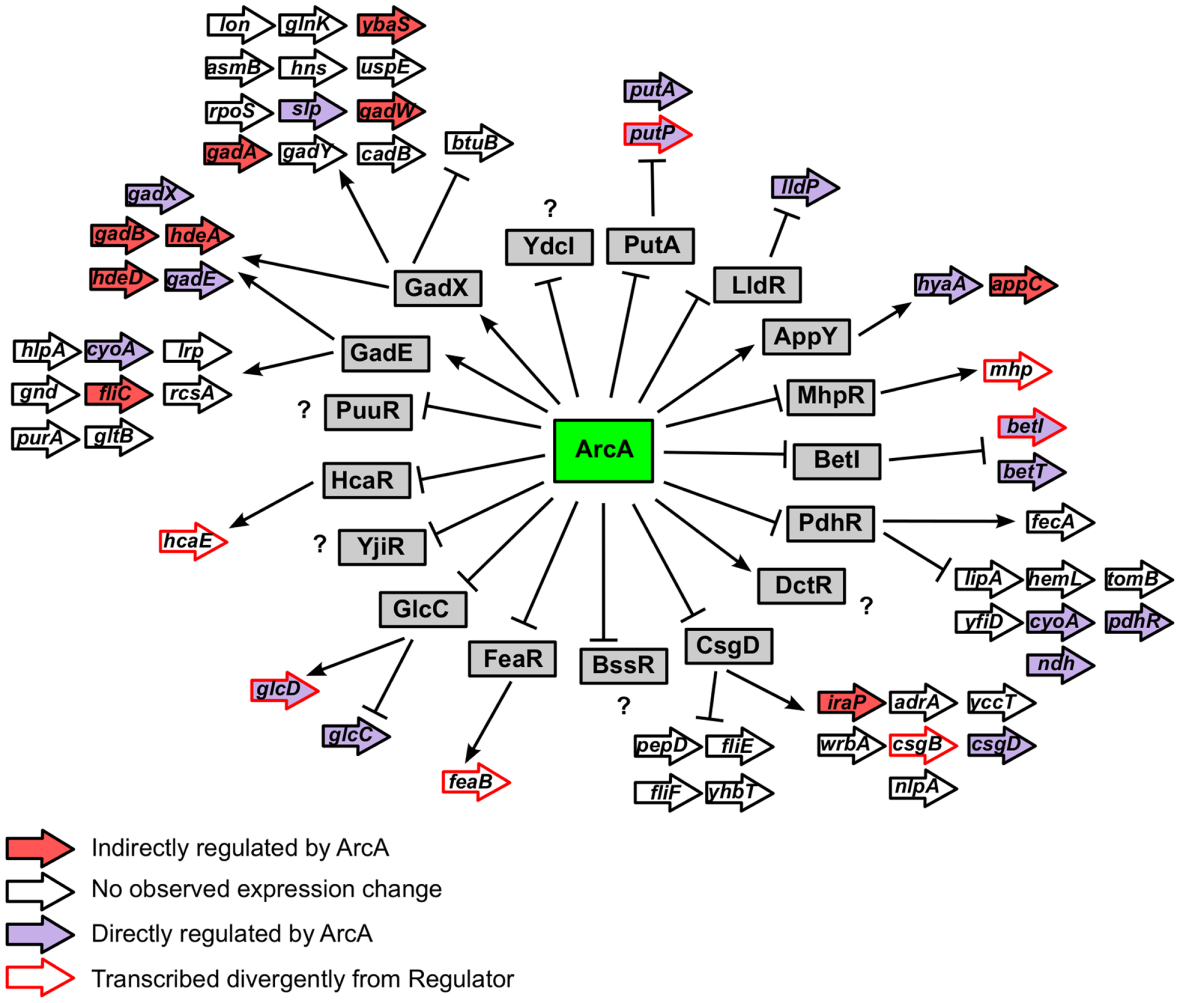
Hierarchical mode of ArcA-mediated transcriptional regulation. Depicts the hierarchical mode of transcriptional control mediated by ArcA. The 17 transcription factors under direct ArcA control are indicated by grey boxes. Operons under the control of each transcription factor (as annotated in EcoCyc [47]) are depicted with arrows and colored based on ArcA regulation as indicated in the legend. Only the first gene of every operon is labeled.

Of the 229 operons regulated by ArcA, 145 lacked ArcA binding *in vivo* and have not been shown previously to be directly regulated by ArcA. To assess whether an ArcA binding site was missed by our ChIP analyses at any of these operons, we searched the intergenic region upstream of each operon using a cutoff of 15 bits (representing the average sequence conservation of the ArcA sequence logo). An ArcA binding site was identified upstream of only seven operons (*acnA*, *prpR*, *folE*, *yibF*, *yigI*, *dcuC/crcA*), indicating that the remaining 135 operons are likely regulated through an indirect mechanism. Since ArcA directly regulates the expression of 17 transcription factors, a hierarchical mode of regulation could, in part, explain the differential expression of some of these operons. Although not all of these transcription factors are expected or known to be active under our growth conditions, differential expression of nine operons can likely be traced to one of these transcription factors (Figure 8). For example, the expression of the AppY dependent *appCBA-yccB* operon [52] is decreased when *arcA* is deleted, presumably because of the decrease in *appY* activation by ArcA. In addition, four target operons (*folE*, *gpmA*, *dld* and *eco*) of the ArcA-activated sRNA, FnrS were upregulated in the *arcA* mutant [57], [58]. Finally, although we did not identify an ArcA binding site upstream of *arcZ*, the downregulation of *sdaC* (the most strongly repressed target of the ArcZ sRNA in *S. enterica* [64]) in the absence of *arcA* is consistent with ArcA-dependent activation of *arcZ* [65].

### ArcA prevents the oxidation of non-fermentable carbon sources during fermentation

Examination of EcoCyc (v15.5) [47] for annotated dehydrogenase enzymes (MultiFun term BC-1), indicated that ArcA either directly or indirectly regulates 37 out of 40 non-glycolytic dehydrogenase enzymes that are favored in the direction of reducing equivalent formation and are not involved in biosynthetic or detoxification functions (Table S9). The carbon oxidation pathways and transporters associated with the substrates of each repressed dehydrogenase are displayed in Figure 6 and the majority of these pathways feed into the TCA cycle for further carbon oxidation. The scope of this repression strongly suggests that a major function of ArcA is to repress all genes encoding enzymes that oxidize non-fermentable carbon compounds, thus preventing the formation of excess reducing equivalents (e.g., NADH, FADH_2_ and quinols) that cannot be readily re-oxidized in the absence of respiration. Nevertheless, despite the extensive upregulation of dehydrogenase enzymes, ArcA mutants have only a small increase in doubling time from 90 to 105 min (Figure S2A) and only a minor alteration in the distribution of fermentation end products (Figure S2B–C). Succinate and ethanol production were marginally increased and decreased by equivalent amounts in a Δ*arcA* strain, respectively, and lactate was not a major fermentation product (Figure S2C). This suggests that the NADH/NAD^+^ ratio was not likely perturbed in our Δ*arcA* strain in agreement with previous results [66], [67].

### Distinct functional roles for ArcA and FNR

Although ArcA and FNR are known to mediate widespread changes in gene expression during the transition from aerobic to anaerobic conditions, the extent of the regulatory overlap between these factors has not been established. Previous gene expression studies have suggested that there may be a large overlap between the genes regulated by ArcA and FNR in both *E. coli* [33] and *S. enterica* [68]. However, comparison of our dataset with that determined recently for FNR using identical growth conditions, suggests that there is little direct coregulation (Figure S3). Of the 37 operons that showed both FNR and ArcA dependent changes in expression, only seven are directly regulated by both ArcA and FNR. Rather, differential expression may result from an indirect effect of a *fnr* deletion on ArcA-P levels, which has been previously suggested to explain the FNR-dependent effect on *sdhC* and *lldP* expression [69]. An additional 12 operons show both ArcA and FNR binding *in vivo* but are differentially expressed in only one dataset (e.g., *focA-pflB, cydAB*). This minimal overlap in the direct regulons of ArcA-P and FNR suggests that these regulators occupy distinct functional roles in anaerobic gene regulation; the ArcA regulon is largely centered around the repression of aerobic carbon oxidation pathways while FNR appears to function as a more general activator of anaerobic gene expression [49]. Some coregulated operons encode enzymes that direct carbon flow towards either oxidative or fermentative metabolism (e.g., *pdhR-aceEF-lpdA*, *focA-pflB*, *yfiD*) while others encode principal components of the respiratory chain (e.g., *nou, ndh, cydA*). However, coregulation of other operons (e.g. *bssR, ompW, ompC, oppA, ygjG, msrB*) by ArcA and FNR is surprising and the physiological implications of this coregulation are unknown.

## Discussion

By comparing ArcA binding *in vivo* with gene expression profiling data, we have greatly expanded the number of operons regulated by ArcA, leading to important insights into the physiological role, mechanism and sequence requirements for ArcA transcriptional regulation. Our analysis indicates that ArcA directly regulates the expression of nearly 100 operons and is predominantly a repressor of genes encoding proteins associated with carbon oxidation pathways. Furthermore, identification of binding sites upstream of many poorly expressed operons (e.g., *paa*) suggests that the direct regulon of ArcA could actually encompass as many as 150 operons. Additionally, our bioinformatic and DNase I footprinting analyses reveal a plasticity in the ArcA binding site architecture that likely has important implications for global regulation of carbon oxidation in *E. coli*.

### ArcA is a global repressor of carbon oxidation pathways

Our finding that under anaerobic conditions, ArcA reprograms metabolism by either directly or indirectly repressing expression of nearly all pathways for carbon sources whose oxidation is coupled to aerobic respiration suggests a global mechanism for NAD^+^ sparing. This strategy would facilitate the preferential oxidation of the fermentable carbon source glucose and the sparing of NAD^+^ for glycolysis by recycling NADH to NAD^+^ via reductive formation of lactic acid, succinate and ethanol. Thus, ATP synthesis via substrate level phosphorylation is ensured and redox balance of NADH/NAD^+^ is maintained during anaerobic glucose fermentation. This function of ArcA exhibits parallels to carbon catabolite repression in that it is another mechanism for selective carbon source utilization in cells. Although carbon catabolite repression preferentially selects for glucose utilization over other sugars, ArcA reinforces glucose catabolism through the repression of non-glycolytic carbon oxidation pathways. By integrating signals from both respiratory and fermentative metabolism, which are both enzymatically linked to the NADH/NAD^+^ redox couple, the ArcAB two component system provides a means for *E. coli* to maintain the NADH/NAD^+^ ratio.

Despite the extensive upregulation of dehydrogenase enzymes in an *arcA* mutant, there was only a minor alteration in fermentation products. This result is in agreement with previous data, which also showed that the NADH/NAD^+^ ratio is not perturbed in strains lacking ArcA during fermentation [66], [67]. The ability of glucose fermenting cells to maintain redox balance in the absence of ArcA likely reflects thermodynamic and kinetic parameters that favor flux via glucose fermentation and the fact that although many dehydrogenases are upregulated, their substrates are not present preventing competition with glycolysis. Indeed, the activity of several dehydrogenases in cellular extracts was previously shown to be increased in an *arcA* mutant. However, the fact that the NADH/NAD^+^ ratio is altered in an *arcB* strain [70] may be explained by the additional roles of ArcB beyond regulating ArcA [39], [71].

Nevertheless, previous studies suggest that ArcA deficiencies may compromise growth more significantly under conditions that more closely parallel the natural habitats of *E. coli*. For example, an *arcA* mutant is defective in both survival during aerobic carbon starvation [72] and in colonization of the mouse intestine [73]. Increased NADH/NAD^+^ ratios have been observed in an *arcA* mutant during microaerobiosis [66], [67], which may contribute to the poor fitness of *arcA* mutants in the gut. Accordingly, it seems reasonable to conclude that this extensive repression of dehydrogenase enzymes by ArcA provides an evolutionary advantage for *E. coli* in its natural habitats where nutrient conditions are in flux and where many more growth substrates (i.e., both carbon sources and electron acceptors) could be encountered.

### ArcA may repress and activate transcription solely by binding DNA

Surprisingly, very little *in vitro* data are available describing mechanisms of ArcA transcription regulation. Nevertheless, the location of the ArcA binding sites and the decrease in σ^70^ occupancy indicate that ArcA represses by occluding RNA polymerase binding like many repressors. However, the mechanism of activation is unlikely to occur through the direct recruitment of RNA Polymerase as observed with ArcA homologs OmpR [60], [61] and PhoB [59] since no conserved location or orientation of ArcA binding sites was evident. Rather, ArcA may increase transcription through an antirepression mechanism. In support of this notion, *in vivo* studies of *hyaA* [31], *cydAB* [74], *appY* [75] and *yfiD* [76] transcription suggest that ArcA activation occurs primarily through disruption of HNS (*cydAB* and *appY*), FNR (*yfiD*) or IscR (*hyaA*) binding. Furthermore, although the mechanism of ArcA activation of *focA-pflB* [77] and the P_Y_ promoter (from the conjugative resistance plasmid R1) [78] is unknown, DNA binding by ArcA alone appears insufficient for its transcriptional activation. In addition, binding of ArcA alone actually repressed transcription of *ndh* [79], despite the observation that *ndh* expression increased when *arcA* was deleted [32]. Although further *in vitro* experiments are necessary to investigate the activation mechanism, it seems plausible that ArcA functions solely by binding DNA and activates only indirectly when its binding interferes with the binding and repression by another transcriptional repressor.

### Plasticity within the architecture of ArcA binding sites

The variation in the number, spacing, location and predicted strength of DR elements within the chromosomal ArcA binding regions suggests plasticity in the architecture of ArcA binding sites for either repressed or activated operons. Although the core of each site is an ArcA box containing two, 11-bp ctc spaced DR elements, the majority of binding sites contain an additional one to three DRs predominantly-spaced by approximately one or two turns of the helix of B-form DNA (11 bp or 22 bp ctc spacing). Multiple DR elements have also been observed for some promoters regulated by OmpR [80] and PhoB [59], [81], [82]. However, it is unclear how pervasive multiple repeat elements are for these regulators because the 41 genomic PhoB binding locations recently mapped by ChIP-chip were not searched for sequence elements beyond a single PhoB Box [83] and a conserved sequence motif was not identified within the majority of the 43 OmpR binding sites identified with ChIP-seq [84]. Although the three direct repeat binding site architecture represents a particularly novel finding for the OmpR/PhoB family of response regulators, at least one other example of a response regulator, ComA in *B. subtilis*, which binds three recognition elements (i.e., an inverted repeat and an additional half site) has been reported and all three elements were shown to be important for both DNA binding and transcriptional activation [85]. Whether the protection of only three DR elements by ArcA reflects binding by a dimer and monomer or two dimers, where the distal subunit is not bound sufficiently to protect sequences from DNase I cleavage, is not yet known.

### Implications of binding site plasticity for global ArcA transcriptional regulation

Since the majority of ArcA binding sites overlap the σ^70^ promoter recognition elements, the plasticity of these cis-regulatory modules may provide an efficient means of encoding binding sites for ArcA, σ^70^-RNAP and perhaps other transcription factors within the same narrow sequence space. We propose that having binding sites with different architectures is also an effective mechanism for producing diverse transcriptional regulatory outputs. First, varying the number, strength or location of DR elements should modulate the extent of anaerobic repression. Second, embedding transcription factor binding sites within an ArcA binding site could either enhance or antagonize ArcA function. For example, the DR elements at the *trxC*, *paaA* and *phoH* promoters also overlap a binding site for a transcriptional activator (CRP for *paaA* [63], OxyR for *trxC* [45]) or a second promoter (P2 at *phoH* [86]), allowing additional regulatory control. Third, sites of varying affinities may also impact the sensitivity of promoters to the phosphorylation state of ArcA. For example, the different binding affinities of DR elements at the *trxC*, *icdA*, *paaA* and *phoH* promoters may allow the fine-tuning of expression in response to changing ArcA-P levels when O_2_ levels vary [16]. Fine tuning of *ompF* and *ompC* expression by OmpR has been observed in response to medium osmolarity due to the presence of multiple upstream OmpR boxes with different affinities [80]. Conversely, the highly cooperative mode of occupancy at the *astC* and *acs* promoters would likely render the expression of these operons exquisitely sensitive to changes in ArcA-P levels; thus, expression may more closely resemble an on-off switch. Ultimately, such flexibility in transcriptional regulatory outputs may be an important means for linking the redox sensing properties of the ArcAB two component system with the global optimization of carbon oxidation pathway levels. Further studies are underway to examine the contribution of different binding site architectures to both DNA binding and transcriptional regulation.

## Materials and Methods

### Growth conditions

All strains were grown in MOPS minimal medium [87] with 0.2% glucose at 37°C and sparged with a gas mix of 95% N_2_ and 5% CO_2_ (anaerobic) or 70% N_2_, 5% CO_2_, and 25% O_2_ (aerobic). Cells were harvested during mid-log growth (OD_600_ of ∼0.3 on a Perkin Elmer Lambda 25 UV/Vis Spectrophotometer).

### Construction of promoter-*lacZ* fusions and β-galactosidase assays

A *paaA* promoter-*lacZ* fusion was constructed as described previously [88] by amplifying the region from +15 to −194 relative to the translation start using primers flanked by XhoI or BamHI restriction sites. A TAA stop codon was incorporated after codon 5 to terminate translation from the Shine-Dalgarno sequence present in this region. The resulting PCR fragment was digested with XhoI and BamHI and directionally cloned into plasmid pPK7035. This *lacZ* promoter construct was then recombined into the chromosomal *lac* operon as previously described [88] to create the *paaA* promoter-*lacZ* fusion and then transduced using P1 *vir* into MG1655 and PK9416 (*ΔarcA*) to creating PK9959 and PK9960 (Table S10). For assays with *paaA*, 1 mM phenylacetic acid (Sigma Aldrich) was added to the minimal glucose media. To terminate cell growth and any further protein synthesis chloramphenicol (final concentration, 20 µg/ml) was added, and cells were placed on ice until assayed for β-galactosidase activity [89]. β-galactosidase values represent the average of at least three replicates.

### Cloning, overexpression and purification of His_6_-ArcA

*arcA* was amplified with primers which incorporated a NheI restriction site, a His_6_-tag and a Tev protease cleavage site (order listed in 5′-3′ direction) on the 5′ end of the gene and a XhoI site at the 3′ end. The NheI and XhoI digested fragments were cloned into plasmid pET 21-d to generate plasmid PK9431 for protein production. E. coli BL21(DE3), containing PK9431 was grown at 37°C until an OD_600_ of 0.5–0.6 was reached then 1 mM isopropyl-1-thio-β-D-galactopyranoside (IPTG) was added. After seven hours at 30°C, cells were harvested, suspended in 5 mM imidazole, 50 mM Tris-Cl, pH 8.3 and 0.3 M NaCl and lysed by sonication. His_6_-ArcA was isolated from cell lysates by passage over a Ni-NTA column pre-equilibrated with 5 mM imidazole, washing extensively with the same buffer followed by 50 mM imidazole, and then eluting with a linear gradient of 50–500 mM imidazole. Fractions containing the overexpressed His_6_-ArcA, determined by electrophoresis, were dialyzed against 50 mM Tris-Cl, pH 8.0 and 0.1 M NaCl and concentrated. Antibodies to ArcA were obtained from Harlan (Indianapolis, In), affinity purified prior to use and determined to be specific to ArcA by Western blot (data not shown). For DNase I footprinting, the His_6_ tag was removed from ArcA by overnight incubation with tobacco etch virus (TEV) protease at 4°C and passage over a Ni^2+^-agarose column (Qiagen). The protein concentration of ArcA (reported here as monomers) was determined with the Coomassie Plus protein assay reagent (Pierce), using bovine serum albumin as a standard.

### Chromatin immunoprecipitation followed by hybridization to a microarray chip or high-throughput sequencing

ChIP was performed as previously described [90] using the affinity purified ArcA polyclonal antibodies. ChIP DNA along with corresponding input DNA were amplified by linker-mediated PCR and labeled with Cy3 or Cy5-random 9-mers then hybridized as previously described [49] to custom-made *E. coli* K-12 MG1655 tiled genome microarrays (Roche NimbleGen, Inc, Madison, WI). The hybridized microarrays were scanned using NimbleGen Hybridization System 4 and the PMT was adjusted as previously described [49]. Quantile normalization (“normalize.quantiles” in the R package VSN) [91] was used to obtain the same empirical distribution across the Cy3 and Cy5 channels and across biological replicate arrays to correct for dye intensity bias and to minimize microarray-to-microarray absolute intensity variations as previously described [92]. The log_2_ of the ratio of experimental signals (Cy5) to control signals (Cy3) was calculated. Regions of the genome enriched for occupancy by ArcA were identified using TAMALPAIS [93] L2 and L3 stringency levels (95th percentile/p<0.0001 and 98th percentile/p<0.05 of the log_2_ ratio for each chip, respectively) with the anaerobic fermentative ArcA data. Only enriched regions that were significant in both biological replicates were considered, resulting in the identification of 194 binding regions. Four false positives were eliminated from the data set by analyzing technical replicate ChIP-chip results from a strain lacking *arcA* (PK9416; Table S11). Fifty-three false positives were eliminated because we found that they resulted from ArcA co-immunoprecipitating with RNA polymerase at highly transcribed regions (Figure S4; Table S12; Text S1; Table S12) leaving 137 regions. The phosphorylation dependence of ArcA DNA binding at these sites was determined by performing a single biological replicate ArcA ChIP-chip experiment under aerobic conditions. For visualization, the anaerobic ArcA biological replicates were averaged then median smoothed using a 300 bp window using MochiView [94].

For ChIP-seq, enriched ChIP DNA from two additional biological replicates from anaerobic ArcA samples were submitted to the University of Wisconsin-Madison DNA Sequencing Facility for library construction and Illumina sequencing performed as previously described [49]. A total of 1,364,908 and 12,074,358 reads were obtained for the ChIP replicates. Greater than 90% and 80% of these reads, respectively, mapped uniquely to the K12 MG1655 genome (version U00096.2) using the software package SOAP release 2.20, allowing no more than two mismatches [95]. The CSDeconv algorithm [41] was then used to determine significantly enriched regions in high resolution using both ChIP-seq replicates and two anaerobic input samples [49] from the same sequencing run as the ArcA ChIP samples. Reads that mapped uniquely within the seven rRNA operon regions were eliminated to allow the algorithm to run more efficiently. CSDeconv was run with Matlab v7.11.0 (R2010b) using the following parameters: LLR = 21.75 and alpha = 800 for replicate one and LLR = 22 and alpha = 550 for replicate two. The find_enriched function was modified to account for differences in sequencing depth between the IP and Input samples. Correction factors of 2.98 (replicate 1) and 0.6579 (replicate 2), calculated by dividing the number of unique reads in the Input sample by the number of reads in the ChIP sample for replicates one and two, respectively, were multiplied by nip and the forward and reverse kernel density calculations for both the forward and reverse strands of the ChIP sample. FDRs of 0.0154 and 0.0156 for replicates one and two, respectively, were calculated by a sample swap (the number of peaks in the Input over the ChIP sample divided by the number of detections in the ChIP over the control sample). From 222 enriched regions generated from two independent ChIP-seq replicates, 146 ArcA-P binding regions (Table S1) were obtained using the same filtering criteria described for ChIP-chip (Table S12; Text S1). For visualization of the ChIP-seq data, the raw tag density at each position was calculated using QuEST version 2.0 [96] and normalized as tag density per million uniquely mapped reads.

The final list of 176 binding regions was obtained by searching binding regions that were found in only one ChIP-seq replicate (48) or were unique to ChIP-chip (28) with the ArcA box PWM (see below) using a cutoff of 10 bits as 99% of ArcA boxes in the alignment have an individual information content of 10 bits or greater. An ArcA binding site was identified in 30 of these binding regions (15 from ChIP-chip and 15 from ChIP-seq) which were, therefore, combined with the 146 regions found in both ChIP-seq replicates to produce the final list of 176 ArcA chromosomal binding regions (Table S1).

### ArcA PWM construction and identification of predicted ArcA binding sites

Based on the improved resolution of ChIP-seq, sequence corresponding to a 200 bp window around each of the 146 CSDeconv binding regions (averages of the two replicates) was searched for a common motif using MEME [42] with the parameters -mod zoops -nmotifs 1 -minw 18 -maxw 25. Using the alignment from MEME, a sequence logo was built using the Delila software package with the delila, encode, rseq, dalvec, and makelogo programs [97]. A PWM generated from this alignment was used to search the 146 binding regions with a cutoff of 9 bits as this represents the lowest scoring ArcA box included in the MEME alignment. Using the program localbest, only the best scoring ArcA box within a 200 bp region was retained due to several instances of overlapping ArcA-P boxes being identified (sites with three and four DR elements). The resulting 128 ArcA-P boxes were used to make the final sequence logo (Figure 3A). The delila program ri [97] was used to calculate the information content of individual sequences within the positions −3 and 14, which ranged from 9.1 to 21 bits (Table S3). A PWM derived from the conservation of bases between positions −3 and 14 in these 128 ArcA-P boxes, is referred to throughout the paper as the ArcA box PWM. No unique motif was identified within the 18 binding regions without a match to the ArcA box. The scan program [97] was used to search DNA sequences upstream of differentially expressed operons that were not enriched in ChIP using the ArcA box PWM. The *E. coli* K12 genome sequence [98] was obtained from GenBank (v. U00096.2) and a bit score cutoff of 15 bp bits was used as this represents the average information content of the ArcA box PWM. The localbest program was used to select the best scoring ArcA box within a 200 bp region in cases where two sites were predicted in close proximity.

To construct the 10 bp PWM corresponding to a single direct repeat element, positions −3 to 6 and 8 to 17 from the 128 sequences used to make the ArcA box sequence logo were aligned as they correspond to the nucleotides contacted by each PhoB monomer in the crystal structure of the C-terminus of PhoB bound to its PhoB box [99]. Due to the identical spacing between DR elements and the highly similar nucleotide compositions of the PhoB and ArcA boxes, this structure likely serves as a good model for the nucleotides contacted by each ArcA monomer. A bit score cutoff of 0, which represents the theoretical lowest limit of binding [97], was used to search a 100 bp region surrounding each identified ArcA box with the scan program to identify sites with additional repeat elements. Where displayed, sequence walkers were used to visualize matches to the ArcA-P binding site using the lister program [100].

### Gene expression profiling with a microarray

An in-frame *ΔarcA* deletion strain was constructed by replacing the coding region of *arcA* (codons 2–238) with a Cm^R^ resistance cassette flanked by FLP recognition target (FRT) sites from plasmid pKD32 in strain BW25993/pKD46, as described previously [101] to generate PK7510. Transduction with P1 *vir* was used to move the *arcA::cat* allele into MG1655 to produce PK7514. The Cm^R^ cassette of PK7514 was removed by transforming this strain with pCP20-encoding FLP recombinase [101] then screening for loss of Cm, generating PK9416 (Table S10). The deletion was confirmed by sequencing.

RNA was isolated from triplicate MG1655 and Δ*arcA* (PK9416) strains using a hot-phenol method [102]. The RNA was reverse transcribed to cDNA, labeled with Cy3-random 9-mers and hybridized onto the Roche NimbleGen *E. coli* 4plex Expression Array Platform (4×72,000 probes, Catalog Number A6697-00-01) as previously described [49]. The expression data was normalized using Robust Multi-Array (RMA) [103] and statistical analysis was performed with Arraystar III software (DNASTAR). Transcripts exhibiting a statistically significant (moderated t-test p-value<0.05) change in expression greater than 2-fold were considered differentially expressed and grouped into operons using operon definitions in EcoCyc [47] if at least two of the genes in a particular operon exhibited differential expression.

### End product analysis

Samples (2 ml) for end product analysis were collected during log phase, the transition to stationary phase and in stationary phase (Figure 7A). Cells were removed by passage through a 0.2 µm filter and the supernatant was stored at −80°C prior to analysis. For each sample, glucose, pyruvic acid, succinic acid, lactic acid, formic acid, acetic acid, and ethanol were separated by high-performance liquid chromatography (HPLC) and subsequently quantified as previously described [104].

### DNase I footprinting

Plasmids containing predicted ArcA-P binding sites were generated by PCR amplification of chromosomal DNA with primers flanked by XhoI or BamHI restriction sites and cloned into pPK7179 or pPK7035 (for the *icdA* promoter)(Table S10). The positions of the promoter fragments relative to the previously identified transcription start sites are as follows: for icdA [23], −216 to +65; for *acs* (*P_2_*) [105], −172 to +44; for *phoH* (P_2_) [86], −161 to +20; for *paaA* [106], −132 to +55; for *astC* [107], −166 to +62; for *putP* (P_1_) [108], −120 to +56; for *trxC* [45], −118 to +50 ; for *dctA* [109], −185 to +32. The *icdA* fragment contains two promoters: one whose expression is dependent on ArcA (P_1_) and a second promoter whose expression is dependent on FruR (P_2_) [23], [110]. To examine *icdA* expression from only P_1_ in future expression analyses, transcription from P_2_ was eliminated using the site-directed mutagenesis protocol described in [111] to mutate the −10 site from cattat to cggtga. DNA fragments were isolated from pPK7179 or pPK9476 (*icdA*) after digestion with XhoI and BamHI, radiolabelled at the 3′ BamHI end with [α-^32^P]-dGTP (PerkinElmer) and Sequenase Version 2.0 (USB Scientific), isolated from a non-denaturing 5% acrylamide gel and subsequently purified with elutip-d columns (Schleicher and Schuell). ArcA was phosphorylated by incubating with 50 mM disodium carbamyl phosphate (Sigma Aldrich) in 50 mM Tris, pH 7.9, 150 mM NaCl, and 10 mM MgCl2 for 1 h at 30°C [24] and immediately used in the binding assays. Footprinting assays were performed by incubating phosphorylated ArcA with labeled DNA (∼5 nM) for 10 min at 30°C in 40 mM Tris (pH 7.9), 30 mM KCl, 100 µg/ml BSA and 1 mM DTT followed by the addition of 2 µg/ml DNase I (Worthington) for 30 s. The DNase I reaction was terminated by the addition of sodium acetate and EDTA to final concentrations of 300 mM and 20 mM, respectively. The reaction mix was ethanol precipitated, resuspended in urea loading dye, heated for 60 s at 90°C, and loaded onto a 7 M urea, 8% polyacrylamide gel in 0.5× TBE buffer. An A+G ladder was made by formic acid modification of the radiolabeled DNA, followed by piperidine cleavage [112]. The reaction products were visualized by phosphorimaging.

### Data deposition

All genome-wide data from this publication have been deposited in NCBI's Gene Expression Omnibus (GSE46415.

## Supporting Information

Figure S1 Average sequence conservation of binding sites with two, three and four equally spaced DR elements. Sequence logos for DR elements in sites with two (66 sites), three (27 sites) and four (14 sites) equally spaced DR elements were constructed by alignment of each DR element within these binding sites. The sequence conservation (bits) is depicted by the height of the letters with the relative frequencies of each base depicted by its relative heights. The two, three and four DR element binding sites used in this figure are listed in Table S4.
(EPS)

Figure S2 Measurement of fermentation end products in WT and *ΔarcA* strains. Fermentation end product analysis for MG1655 (filled symbols) and isogenic MG1655 *ΔarcA* (open symbols) strains during log phase, the transition to stationary phase and stationary phase. (A) The growth curves for both strains with the sampling points indicating by red diamonds. (B) Concentration of acetate, formate and glucose. (C) Concentration of succinate, ethanol and lactate. Symbols are described in the legend and error bars represent the standard deviation of three biological replicates.
(EPS)

Figure S3 Comparison of the ArcA and FNR direct regulons. Overlap between the direct regulons of ArcA and FNR [49], determined from cells grown under anaerobic conditions with glucose as a carbon source. Repression denoted by (−) and activation by denoted by (+).
(EPS)

Figure S4 Correlation between ArcA and RNAP at regions of high RNAP occupancy. ArcA appears to crosslink with RNAP at regions of high RNAP occupancy in a phosphorylation independent manner. (A) Anaerobic and aerobic ChIP-chip enrichment of ArcA (blue and cyan, respectively) across an entire ribosomal operon mirrors the RNAP β signal. This signal is largely reduced when the ChIP-chip experiment was performed in an Δ*arcA* strain (red). (B) Correlation of the anaerobic WT (blue) or Δ*arcA* (red) ChIP-chip signal with that for RNAP β. To construct this plot, the genome was divided into 300 bp non-overlapping bins and the maximum log_2_ ratio was extracted for each sample in each bin. The solid lines represent the regression lines for each data set for RNAP β log_2_ ratios greater than or equal to 1.75 with the corresponding Pearson correlation coefficient (r) indicated in the figure legend. (C) Correlation of the aerobic (cyan) and anaerobic (blue) ArcA ChIP-chip signal with that for RNAP beta performed as described for B. (D) Maximal aerobic or anaerobic ArcA log_2_ ratios within all 137 enriched regions that were retained in the ArcA dataset (Table S10). (E) Maximal aerobic or anaerobic ArcA log_2_ ratio within all 53 enriched regions that were eliminated from the ArcA dataset due to ArcA likely crosslinking with RNAP (Table S9).
(EPS)

Table S1 176 ArcA binding regions identified with ChIP-seq and ChIP-chip.
(XLS)

Table S2 ChIP-chip enriched regions resolved into multiple binding regions with ChIP-seq.
(XLS)

Table S3 128 ArcA boxes used to build ArcA box sequence logo in Figure 3A.
(XLS)

Table S4 Sites with multiple predicted DR elements.
(XLS)

Table S5 229 operons that are differentially expressed in an Δ*arcA* strain compared to WT.
(XLS)

Table S6 85 operons directly regulated by ArcA.
(XLS)

Table S7 Operons with an upstream ArcA binding region that exhibit an ArcA-dependent change in expression in other studies but not under our growth conditions.
(XLS)

Table S8 31 operons with an upstream ArcA binding region that are lowly expressed.
(XLS)

Table S9 Dehydrogenases that are directly or indirectly regulated by ArcA.
(XLS)

Table S10 Strains and plasmids used in this study.
(XLS)

Table S11 ArcA ChIP-chip and ChIP-seq peaks filtered due to enrichment in Δ*arcA* strain.
(XLS)

Table S12 ArcA ChIP-chip and ChIP-seq peaks filtered due to crosslinking with RNAP.
(XLS)

Text S1 File containing supporting methods and references for the information found in the supporting tables.
(DOC)
