# Supplementary material for: The Bacterial Response Regulator ArcA Uses a Diverse Binding Site Architecture to Regulate Carbon Oxidation Globally
Source: PLoS Genet. 2013 Oct 17;9(10):e1003839. doi: 10.1371/journal.pgen.1003839 (PMC3798270; doi:10.1371/journal.pgen.1003839)
Supplement: Text S1 — File containing supporting methods and references for the information found in the supporting tables. (DOC) [file pgen.1003839.s017.doc]

**Supplemental Methods and References**

**The bacterial response regulator ArcA uses a diverse binding site architecture to regulate carbon oxidation globally**

Dan M. Park, Md. Sohail Akhtar, Aseem Z. Ansari, Robert Landick, Patricia J. Kiley

**Supplemental Methods**

**ChIP-chip and ChIP-seq peak filtering**

As a first step to eliminate false positive enriched regions, the maximum median smoothed log_2_ ratio within each TAMALPAIS [1] defined enriched region was compared for the anaerobic fermentative ArcA and Δ*arcA* ChIp-chip samples. The four enriched regions where the Δ*arcA* log_2_ ratio was greater than 60% of the ArcA value were eliminated from both ChIP-chip and ChIP-seq datasets (Table S11).

Additionally, upon visual inspection of the ChIP-chip data, it was apparent that a subset of peaks were unexpectedly located in regions of high RNA polymerase (RNAP) occupancy [2], such as genes encoding tRNAs, rRNA and ribosomal proteins. These peaks were distinct from the majority of identified ArcA peaks since a putative ArcA binding site could not be identified in any of these regions when searched with an ArcA~P box PWM using a cutoff of 13 bits. Second, the ArcA signal correlated with the RNAP beta subunit ChIP-chip signal at regions of high RNAP occupancy (r = 0.71, p = 4.96E-147; Figure S4B), which was most striking at ribosomal operons, where the ArcA signal extends across the entire length of the operon (Figure S4A). This signal was dependent on ArcA as it was significantly reduced in the control ChIP-chip experiment in a strain lacking ArcA (Figure S4B). Third, results from an ArcA ChIP-chip experiment performed under aerobic growth conditions indicated that this elevated signal is independent of the phosphorylation state of ArcA as a strong correlation (r^2^ = .81, p = 1.81E-166 Figure S4C) between ArcA and RNAP was still evident. In contrast, a clear reduction in ArcA occupancy was observed under aerobic conditions for nearly all retained binding regions (Figure S4D-E). Thus, we concluded that ArcA was crosslinking with RNAP at highly transcribed regions rather than directly binding DNA.

To remove these false positive regions, due to ArcA crosslinking with RNAP, we used the following three filtering steps 1) The log_2_ ratios for two RNA Polymerase subunit β ChIP-chip replicates [2] were quantile normalized, averaged, then median smoothed using a 300 bp window [3]. The maximum β log_2_IP/Input value within a 100bp region surrounding the coordinate of the maximal anaerobic ArcA log_2_IP/Input (median smoothed with 300 bp window) within each TAMALPAIS-defined enriched region was extracted. If this value was in the top 95th percentile of β log_2_IP/Input the ArcA peak region was flagged. 2) Next, we performed the same calculation with the median smoothed log_2_ ratios for the aerobic ArcA ChIP-chip data and compared this value with corresponding value for the anaerobic ArcA ChIP-chip data for all flagged regions. Regions where the aerobic ArcA value was greater than 70% of the ArcA value remained flagged while those below 70% were retained in the dataset. 3) Finally, the sequences corresponding to the flagged enriched regions were extracted and searched with the ArcA box PWM using a cutoff of 13 bits. Flagged regions without a predicted ArcA box were eliminated (53 regions; Table S12) leaving 137 ChIP-chip regions. Eliminated regions primarily corresponded to tRNA, rRNA and ribosomal protein encoding regions (Table S12). Although 20 of the eliminated binding regions were upstream of genes, none exhibited an ArcA-dependent change in gene expression (data not shown), suggesting that ArcA enrichment at these locations was not affecting transcription of known genes under anaerobic conditions. Furthermore, β-galactosidase assays with *rrnBp1* and *rrnBp2* promoter*-lacZ* fusions [4,5] demonstrated a lack of an *arcA* effect on ribosomal operon expression (data not shown).

All ChIP-seq peaks corresponding to the filtered ChIP-chip peaks were removed from the ChIP-seq data set (Figure S12). In cases where significant ArcA enrichment was not detected in the anaerobic ChIP-chip dataset by TAMALPAIS [1], a 100 bp sequence window surrounding the binding location detected in the ChIP-seq dataset by CSDeconv [6] was used to extract the maximal β, anaerobic ArcA, and aerobic ArcA ChIP-chip log_2_ ratio values and the same filtering criteria was applied to filter peaks (Figure S12).

**Association of ArcA binding regions with operons**

The 176 ArcA binding regions were associated with specific operons using the operon annotations in EcoCyc [7]. To accomplish this, the 176 binding regions were first grouped based on their location in intergenic (162) or intragenic (14) regions. For the simplest case where a single ArcA binding region was located within an intergenic region (134/162 binding regions), the ArcA binding region was associated with a particular operon if it was located upstream of that gene. If this was a divergently transcribed region, the ArcA binding region was associated with both operons as ArcA binding could affect expression of either operon. For the 13 intergenic locations with multiple ArcA binding regions (28 binding regions total), seven were located within divergently transcribed regions so a single ArcA binding region was tentatively associated with each divergent operon (e.g., *sdhC/gltA* region). However, it is possible that both binding regions regulate transcription of the same operon. For the six non-divergent locations, all binding regions were associated with the downstream operon (e.g., *cydAB*). In total, 159 of the 162 intergenic binding regions were linked with genes while three were located downstream of oppositely oriented genes. Finally, the 14 intragenic binding regions were not initially linked with specific genes but were retained in the dataset as they could affect transcription from an intragenic promoter or a downstream intergenic promoter. In fact, 4 such binding regions were upstream of a differentially expressed gene (Table S6).

# References

1. Bieda M, Xu X, Singer MA, Green R, Farnham PJ (2006) Unbiased location analysis of E2F1-binding sites suggests a widespread role for E2F1 in the human genome. Genome Res 16: 595-605.

2. Myers K, Yan H, Ong IM, Chung D, Liang K, et al. (2013) Genome-scale Analysis of *E. coli* FNR Reveals Complex Features of Transcription Factor Binding. PLoS Genet 9:e1003565.

3. Homann OR, Johnson AD (2010) MochiView: versatile software for genome browsing and DNA motif analysis. BMC Biol 8: 49.

4. Hirvonen CA, Ross W, Wozniak CE, Marasco E, Anthony JR, et al. (2001) Contributions of UP elements and the transcription factor FIS to expression from the seven *rrn* P1 promoters in *Escherichia coli*. J Bacteriol 183: 6305-6314.

5. Murray HD, Appleman JA, Gourse RL (2003) Regulation of the *Escherichia coli rrnB* P2 promoter. J Bacteriol 185: 28-34.

6. Lun DS, Sherrid A, Weiner B, Sherman DR, Galagan JE (2009) A blind deconvolution approach to high-resolution mapping of transcription factor binding sites from ChIP-seq data. Genome Biol 10: R142.

7. Keseler IM, Collado-Vides J, Santos-Zavaleta A, Peralta-Gil M, Gama-Castro S, et al. (2011) EcoCyc: a comprehensive database of *Escherichia coli* biology. Nucleic Acids Res 39: D583-590.

8. Kim D, Hong JS, Qiu Y, Nagarajan H, Seo JH, et al. (2012) Comparative analysis of regulatory elements between *Escherichia coli* and *Klebsiella pneumoniae* by genome-wide transcription start site profiling. PLoS Genet 8: e1002867.

9. Durand S, Storz G (2010) Reprogramming of anaerobic metabolism by the FnrS small RNA. Mol Microbiol 75: 1215-1231.

10. Iuchi S, Cole ST, Lin EC (1990) Multiple regulatory elements for the *glpA* operon encoding anaerobic glycerol-3-phosphate dehydrogenase and the *glpD* operon encoding aerobic glycerol-3-phosphate dehydrogenase in *Escherichia coli*: further characterization of respiratory control. J Bacteriol 172: 179-184.

11. Kim SJ, Han YH, Kim IH, Kim HK (1999) Involvement of ArcA and Fnr in expression of *Escherichia coli* thiol peroxidase gene. IUBMB Life 48: 215-218.

12. Lynch AS, Lin EC (1996) Transcriptional control mediated by the ArcA two-component response regulator protein of *Escherichia coli*: characterization of DNA binding at target promoters. J Bacteriol 178: 6238-6249.

13. Liu X, De Wulf P (2004) Probing the ArcA-P modulon of *Escherichia coli* by whole genome transcriptional analysis and sequence recognition profiling. J Biol Chem 279: 12588-12597.

14. Shalel-Levanon S, San KY, Bennett GN (2005) Effect of oxygen, and ArcA and FNR regulators on the expression of genes related to the electron transfer chain and the TCA cycle in *Escherichia coli*. Metab Eng 7: 364-374.

15. Drapal N, Sawers G (1995) Purification of ArcA and analysis of its specific interaction with the *pfl* promoter-regulatory region. Mol Microbiol 16: 597-607.

16. Cunningham L, Georgellis D, Green J, Guest JR (1998) Co-regulation of lipoamide dehydrogenase and 2-oxoglutarate dehydrogenase synthesis in *Escherichia coli*: characterisation of an ArcA binding site in the *lpd* promoter. FEMS Microbiol Lett 169: 403-408.

17. Salmon KA, Hung SP, Steffen NR, Krupp R, Baldi P, et al. (2005) Global gene expression profiling in *Escherichia coli* K12: effects of oxygen availability and ArcA. J Biol Chem 280: 15084-15096.

18. Cho BK, Knight EM, Palsson BO (2006) Transcriptional regulation of the *fad* regulon genes of *Escherichia coli* by ArcA. Microbiology 152: 2207-2219.

19. Lamark T, Rokenes TP, McDougall J, Strom AR (1996) The complex *bet* promoters of *Escherichia coli*: regulation by oxygen (ArcA), choline (BetI), and osmotic stress. J Bacteriol 178: 1655-1662.

20. Cotter PA, Gunsalus RP (1992) Contribution of the *fnr* and *arcA* gene products in coordinate regulation of cytochrome o and d oxidase (*cyoABCDE* and *cydAB*) genes in *Escherichia coli*. FEMS Microbiol Lett 70: 31-36.

21. Park SJ, McCabe J, Turna J, Gunsalus RP (1994) Regulation of the citrate synthase (*gltA*) gene of *Escherichia coli* in response to anaerobiosis and carbon supply: role of the *arcA* gene product. J Bacteriol 176: 5086-5092.

22. Iuchi S, Lin EC (1988) *arcA* (*dye*), a global regulatory gene in *Escherichia coli* mediating repression of enzymes in aerobic pathways. Proc Natl Acad Sci U S A 85: 1888-1892.

23. Shen J, Gunsalus RP (1997) Role of multiple ArcA recognition sites in anaerobic regulation of succinate dehydrogenase (*sdhCDAB*) gene expression in *Escherichia coli*. Mol Microbiol 26: 223-236.

24. Park SJ, Tseng CP, Gunsalus RP (1995) Regulation of succinate dehydrogenase (*sdhCDAB*) operon expression in *Escherichia coli* in response to carbon supply and anaerobiosis: role of ArcA and Fnr. Mol Microbiol 15: 473-482.

25. Park SJ, Chao G, Gunsalus RP (1997) Aerobic regulation of the *sucABCD* genes of *Escherichia coli*, which encode alpha-ketoglutarate dehydrogenase and succinyl coenzyme A synthetase: roles of ArcA, Fnr, and the upstream *sdhCDAB* promoter. J Bacteriol 179: 4138-4142.

26. Chao G, Shen J, Tseng CP, Park SJ, Gunsalus RP (1997) Aerobic regulation of isocitrate dehydrogenase gene (*icd*) expression in *Escherichia coli* by the *arcA* and *fnr* gene products. J Bacteriol 179: 4299-4304.

27. Partridge JD, Scott C, Tang Y, Poole RK, Green J (2006) *Escherichia coli* transcriptome dynamics during the transition from anaerobic to aerobic conditions. J Biol Chem 281: 27806-27815.

28. Pellicer MT, Lynch AS, De Wulf P, Boyd D, Aguilar J, et al. (1999) A mutational study of the ArcA-P binding sequences in the *aldA* promoter of *Escherichia coli*. Mol Gen Genet 261: 170-176.

29. Park SJ, Gunsalus RP (1995) Oxygen, iron, carbon, and superoxide control of the fumarase *fumA* and *fumC* genes of *Escherichia coli*: role of the *arcA*, *fnr*, and *soxR* gene products. J Bacteriol 177: 6255-6262.

30. van der Rest ME, Frank C, Molenaar D (2000) Functions of the membrane-associated and cytoplasmic malate dehydrogenases in the citric acid cycle of *Escherichia coli*. J Bacteriol 182: 6892-6899.

31. Pellicer MT, Fernandez C, Badia J, Aguilar J, Lin EC, et al. (1999) Cross-induction of *glc* and *ace* operons of *Escherichia coli* attributable to pathway intersection. Characterization of the *glc* promoter. J Biol Chem 274: 1745-1752.

32. Feng Y, Cronan JE (2010) Overlapping repressor binding sites result in additive regulation of *Escherichia coli* FadH by FadR and ArcA. J Bacteriol 192: 4289-4299.

33. Park SJ, Cotter PA, Gunsalus RP (1995) Regulation of malate dehydrogenase (*mdh*) gene expression in *Escherichia coli* in response to oxygen, carbon, and heme availability. J Bacteriol 177: 6652-6656.

34. Davies SJ, Golby P, Omrani D, Broad SA, Harrington VL, et al. (1999) Inactivation and regulation of the aerobic C(4)-dicarboxylate transport (*dctA*) gene of *Escherichia coli*. J Bacteriol 181: 5624-5635.

35. Nesbit AD, Fleischhacker AS, Teter SJ, Kiley PJ (2012) ArcA and AppY antagonize IscR repression of hydrogenase-1 expression under anaerobic conditions, revealing a novel mode of O_2_ regulation of gene expression in *Escherichia coli*. J Bacteriol 194: 6892-6899.

36. Zhang Y, Xiao M, Horiyama T, Li X, Nishino K, et al. (2011) The multidrug efflux pump MdtEF protects against nitrosative damage during the anaerobic respiration in *Escherichia coli*. J Biol Chem 286: 26576-26584.

37. Brondsted L, Atlung T (1994) Anaerobic regulation of the hydrogenase 1 (*hya*) operon of *Escherichia coli*. J Bacteriol 176: 5423-5428.

38. Richard DJ, Sawers G, Sargent F, McWalter L, Boxer DH (1999) Transcriptional regulation in response to oxygen and nitrate of the operons encoding the [NiFe] hydrogenases 1 and 2 of *Escherichia coli*. Microbiology 145 ( Pt 10): 2903-2912.

39. Brondsted L, Atlung T (1996) Effect of growth conditions on expression of the acid phosphatase (*cyx-appA*) operon and the *appY* gene, which encodes a transcriptional activator of *Escherichia coli*. J Bacteriol 178: 1556-1564.

40. Turlin E, Sismeiro O, Le Caer JP, Labas V, Danchin A, et al. (2005) 3-phenylpropionate catabolism and the *Escherichia coli* oxidative stress response. Res Microbiol 156: 312-321.

41. Cunningham L, Gruer MJ, Guest JR (1997) Transcriptional regulation of the aconitase genes (*acnA* and *acnB*) of *Escherichia coli*. Microbiology 143 ( Pt 12): 3795-3805.

42. Wyborn NR, Messenger SL, Henderson RA, Sawers G, Roberts RE, et al. (2002) Expression of the *Escherichia coli yfiD* gene responds to intracellular pH and reduces the accumulation of acidic metabolic end products. Microbiology 148: 1015-1026.

43. Rolfe MD, Ocone A, Stapleton MR, Hall S, Trotter EW, et al. (2012) Systems analysis of transcription factor activities in environments with stable and dynamic oxygen concentrations. Open Biol 2: 120091.

44. Kwon O, Druce-Hoffman M, Meganathan R (2005) Regulation of the ubiquinone (coenzyme Q) biosynthetic genes *ubiCA* in *Escherichia coli*. Curr Microbiol 50: 180-189.

45. Datsenko KA, Wanner BL (2000) One-step inactivation of chromosomal genes in *Escherichia coli* K-12 using PCR products. Proc Natl Acad Sci U S A 97: 6640-6645.

46. Kang Y, Weber KD, Qiu Y, Kiley PJ, Blattner FR (2005) Genome-wide expression analysis indicates that FNR of *Escherichia coli* K-12 regulates a large number of genes of unknown function. J Bacteriol 187: 1135-1160.
